# Grazing-incidence optical magnetic recording with super-resolution

**DOI:** 10.3762/bjnano.8.4

**Published:** 2017-01-04

**Authors:** Gunther Scheunert, Sidney R Cohen, René Kullock, Ryan McCarron, Katya Rechev, Ifat Kaplan-Ashiri, Ora Bitton, Paul Dawson, Bert Hecht, Dan Oron

**Affiliations:** 1 Department of Physics of Complex Systems, Weizmann Institute of Science, Rehovot 76100, Israel; 2 Department of Chemical Research Support, Weizmann Institute of Science, Rehovot 76100, Israel; 3 Department of Physics, University of Würzburg, 97070 Würzburg, Germany; 4 Centre for Nanostructured Media, School of Mathematics and Physics, Queen’s University Belfast, Belfast BT7 1NN, UK

**Keywords:** laser absorption, laser heating, thermally assisted magnetic recording

## Abstract

Heat-assisted magnetic recording (HAMR) is often considered the next major step in the storage industry: it is predicted to increase the storage capacity, the read/write speed and the data lifetime of future hard disk drives. However, despite more than a decade of development work, the reliability is still a prime concern. Featuring an inherently fragile surface-plasmon resonator as a highly localized heat source, as part of a near-field transducer (NFT), the current industry concepts still fail to deliver drives with sufficient lifetime. This study presents a method to aid conventional NFT-designs by additional grazing-incidence laser illumination, which may open an alternative route to high-durability HAMR. Magnetic switching is demonstrated on consumer-grade CoCrPt perpendicular magnetic recording media using a green and a near-infrared diode laser. Sub-500 nm magnetic features are written in the absence of a NFT in a moderate bias field of only μ_0_*H* = 0.3 T with individual laser pulses of 40 mW power and 50 ns duration with a laser spot size of 3 μm (short axis) at the sample surface – six times larger than the magnetic features. Herein, the presence of a nanoscopic object, i.e., the tip of an atomic force microscope in the focus of the laser at the sample surface, has no impact on the recorded magnetic features – thus suggesting full compatibility with NFT-HAMR.

## Introduction

Heat-assisted magnetic recording (HAMR) is widely considered the most promising future candidate for achieving some major goals in magnetic storage technology [1–2]. Being mainly developed to increase the storage capacity beyond current limitation predictions of around 1 Tb/in^2^ in perpendicular magnetic recording (PMR), HAMR is expected to secure the market for cheap and long-term stable data storage on hard disk drives (HDDs) for at least another decade, and in combination with technologies like bit-patterned media, perhaps far beyond [3–5]. In HAMR (also called: thermally assisted magnetic recording) the recording medium is locally heated to lower the required magnetic field for writing pitches on the disk. After the heat dissipates, the magnetic field is switched off and the bit is “frozen” in its new magnetic state. This process is akin to the magnetization of macroscopic permanent magnets, which are heated above their Curie point in a homogeneous magnetic bias field during fabrication to assure the highest possible energy product. At the heart of HAMR devices is a near-field transducer (NFT): a device which consists of a light-capturing unit like a coupling grating, a waveguide structure and a surface plasmon resonator (SPR) positioned in immediate proximity to the recording media to generate a strong near field for highly-localized inductive heating of the recording layer during the writing process [6–7].

However, as pointed out in a series of recent studies on the thermal stability of these NFTs [8–11], they are inherently sensitive to thermal degradation under the suggested operating conditions, and current SPR designs might not be able to satisfy the requirement of highly reliable HDDs which have to operate for several years under high load. In addition, current designs feature a magnetic writing tip being laterally displaced by several nanometers from the SPR due to implementation requirements [12], i.e., the recording medium is heated first and only subsequently magnetized. That way, the large thermal gradient due to a SPR cannot be fully exploited.

In this study, we present an alternative design for light delivery, namely by grazing-incidence illumination: the laser is focused on the medium obliquely and absorption is optimized by adjusting the polarization. Thus, a SPR-free HAMR design is possible in which the heating spot (laser focus) overlaps with the magnetic writing tip, rather than being displaced. Using an ordinary commercial HDD platter (featuring PMR CoCrPt recording medium, *T*_C_ ≈ 880 K) we demonstrate, by proper choice of parameters, that even laser powers comparable to SPR-HAMR already cause magnetic switching in the presence of a homogeneous magnetic bias field of μ_0_*H*_B_ = 0.3 T – at a resolution six times smaller than the laser spot size. Two different laser wavelengths were used (532 and 785 nm) to demonstrate the generality of the approach and the experimental results are corroborated by extensive numerical modelling of the heating process and the super-resolution thermal profile.

The first researchers to employ a similar grazing-incidence laser-sample geometry were Guyader and co-workers [13] who studied sub-100 ps all-optical magnetization switching of patterned ferrimagnetic GeFeCo recording material – also in an effort to elude the far-field diffraction limit, but utilizing lateral electric field interference patterns. In contrast to the present study, thermal diffusion played no decisive role due to the sub-ns dynamics.

## Sample Characterization and Experimental Setup

**Figure 1:**
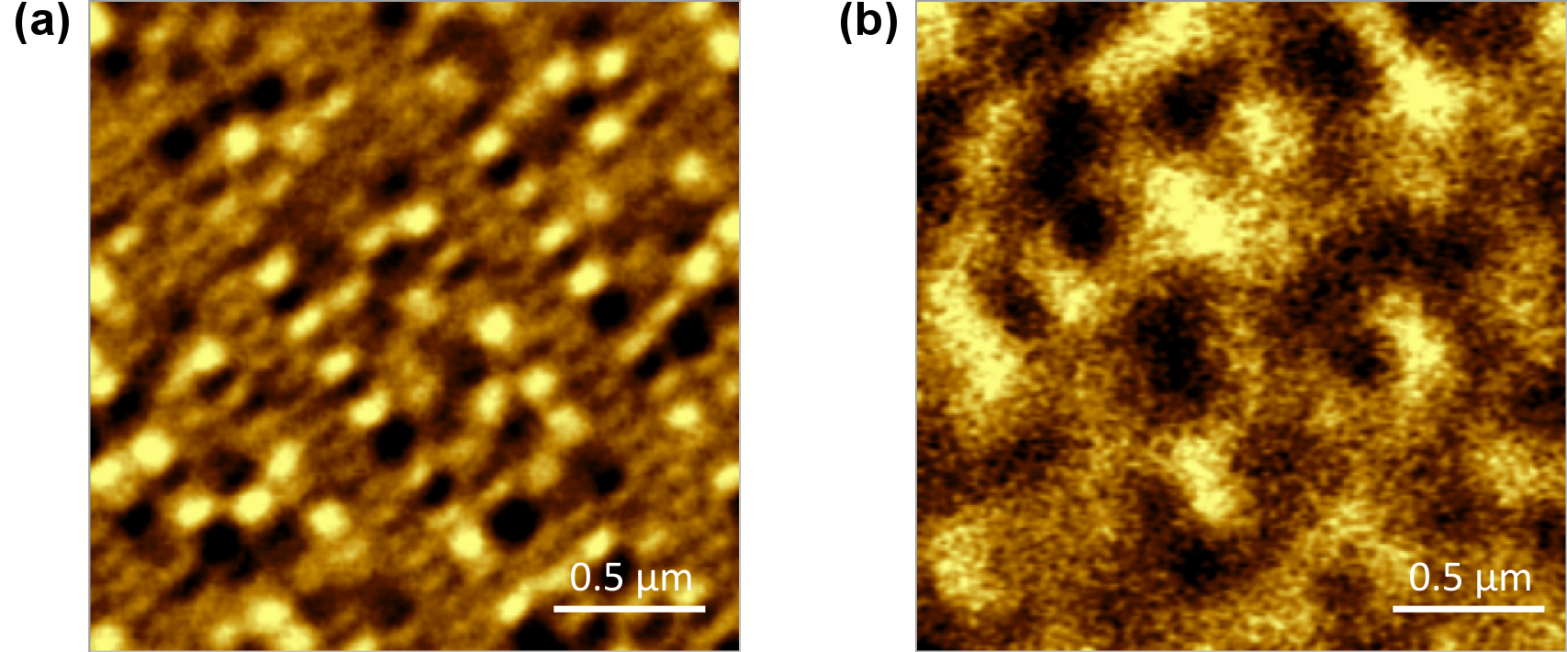
MFM imaging of a HDD featuring PMR with magnetic domains being aligned parallel or antiparallel to the surface normal. (a) Sample piece as is and (b) after exposure to a strong DC magnetic field (μ_0_*H* > 1 T along the surface normal). In the latter case the ordered pitches “broke up” into more or less randomly aligned domains, reminiscent of former tracks. Note, (b) is affected by random noise more severely, with peak intensities being 5 times smaller than in (a).

Grazing-incidence illumination experiments were done on two atomic force microscopes (AFMs): an AIST CombiScope^TM^ and an AIST-NT OmegaScope R^TM^ at wavelengths of 532 nm and 785 nm, respectively. Both provided easy light access at an incident angle of γ = 70° and thus an elliptical laser spot (of around 2 × 6 µm^2^ and 3 × 9 µm^2^ at the sample surface for the 532 nm and the 785 nm laser, respectively). The laser beam diameter was measured optically (scattering on a rough sample) and by following the AIST TERS tip alignment routine (performing an objective scan around the tip and recording the phase signal). This is in agreement with theoretical predictions for the optical setup in use (focal length of focusing objective = 20.3 mm, aperture diameter = 6 mm): for a wavelength of 785 nm the calculated beam waist is *w*_B_ = 3.4 μm and the FWHM of the Gaussian is *w*_FWHM_ = 2.9 μm, assuming a spherical beam under normal incidence (for 532 nm: *w*_B_ = 2.3 μm and *w*_FWHM_ = 2.0 μm). Furthermore, both AFMs were used for magnetic force microscopy (MFM) using Bruker MESP^TM^MFM tips, allowing a resolution of features as small as 50 nm. For magnetic imaging two MFM modes were in use: standard two-pass scans (50 nm lift from the topography measured on the first scan on second pass, 20 nm amplitude) and plane scans performed over a plane surface formed by interpolation of a representative number of points over the scan region and lifted 50 nm from the mean plane height, also performed at 20 nm amplitude. In both cases amplitude and phase signals were recorded, whereas the individual image quality depended on the cantilever resonance frequency setting and the clearer image (phase or amplitude) is presented here. A moving average was applied to all phase/amplitude profiles for better visualization of relevant features. Two different commercial PMR 2.5” HDDs were used for sampling: a Seagate 1 TB/3-platter drive (GoFlex^TM^ series) and a Toshiba 120 GB/1-platter drive. In preparation of laser writing of magnetic features, the platters were exposed to a strong uniform magnetic DC field of μ_0_*H* > 1 T. A comparison of an untreated platter piece and one after field exposure is shown in Figure 1a and Figure 1b, respectively. Although the DC field does not align the magnetization of all grains in the same direction, the surface breaks up into small domains of random order as shown in Figure 1b, and the signal peak intensity of local magnetic features is lowered by more than a factor of 2. This aided in the detection of weaker magnetic signals, with a favorable disposition to stray-fields antiparallel to the DC field, and thus created a more manageable background.

**Figure 2:**
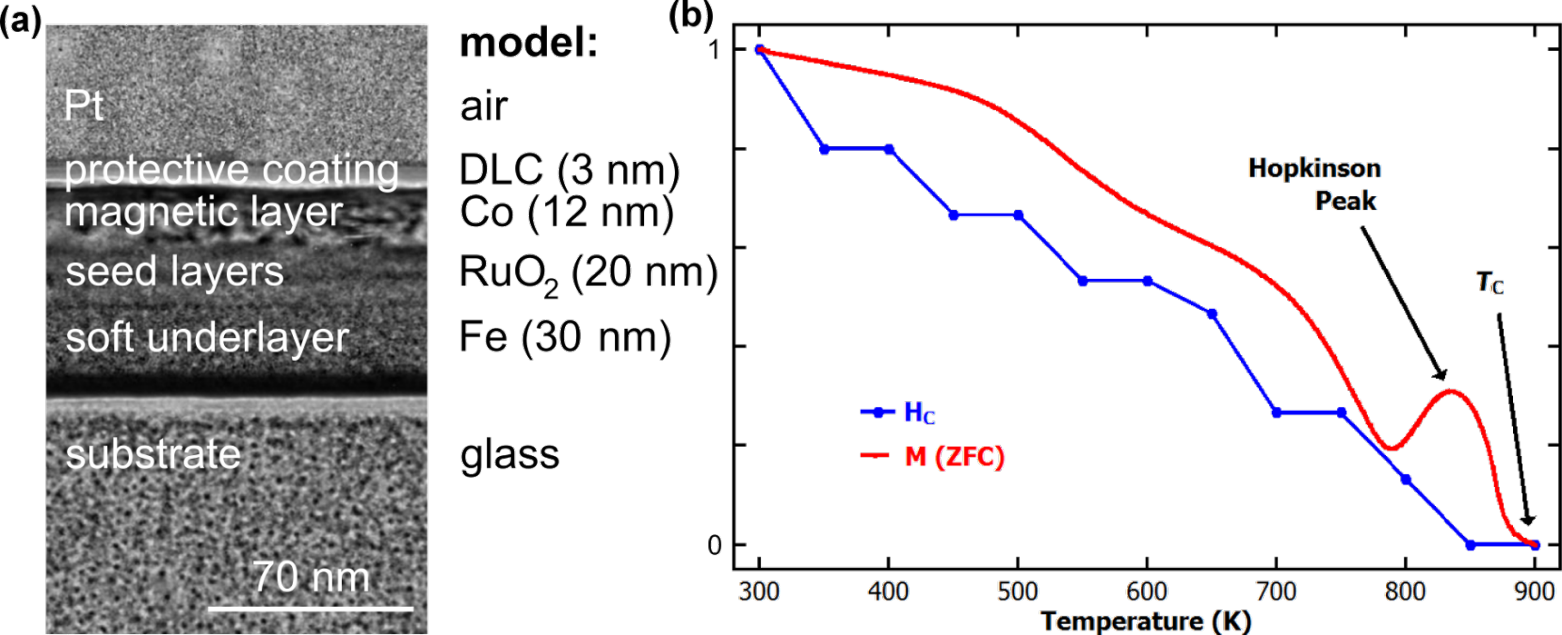
Magnetic recording medium: (a) TEM cross-section image with indication of the stack setup used for modelling. (b) Thermo-magnetic plot of the coercive field strength (blue), normalized to the room temperature value, indicating switching field requirements. Zero-field-cooled (ZFC) thermo-magnetization plot (red; µ_0_*H* = 0.01 T), also normalized to its room temperature value, indicating a Curie temperature of *T*_C_ < 900 K. Note, the thermo-magnetization plot is largely ruled by the magnetization signal of the soft underlayer (SUL), however, at 900 K certainly both, the SUL and the recording layer, became paramagnetic.

Transmission electron microscopy (TEM) cross-sectional imaging was done on a sample lamella prepared via focused ion beam (FIB) of one of the Seagate platters to identify the thin-film layer structure used for modelling as depicted in Figure 2a. It is important to notice the low roughness of the diamond-like carbon (DLC) surface, which is an important prerequisite for high-resolution MFM plane scans. For the electric field and thermal modelling the layer structure was simplified in a sensible manner as indicated in Figure 2a, although the actual layer structure is much more complex, e.g., the recording layer consists of SiO_2_-embedded CoCrPt grains, the seed layer stack contains multiple layers, and the soft underlayer consists of a Fe-rich alloy.

Superconducting quantum interference device (SQUID) magnetometry was used to characterize the magnetic properties of the samples as shown in Figure 2b. The coercivity at room temperature was found to be μ_0_*H*_C_^RT^ ≈ 0.6 T (which is the standard for commercial CoCrPt recording media [14]) and at a temperature of *T*_sw_ ≈ 650 K it dropped to μ_0_*H* = 0.3 T, i.e., less than half its initial value. This critical temperature *T*_sw_ marked the switching threshold, since the applied bias field during illumination was also μ_0_*H*_bias_ = 0.3 T, generated by a strong permanent magnet (FeNdB) beneath the sample with its magnetization parallel or antiparallel to the surface normal (i.e., along the easy axis of the PMR layer grains). For temperatures larger than *T*_sw_ the magnetic features became gradually stronger with respect to the background until saturation. A further temperature increase led to material damage. A main contributor to this gradual improvement is the granularity of the recording layer: depending on their size, the grains have different thermal stability and therefore switch the magnetization direction at different temperatures. The SQUID measurement can only give a coercivity value averaged over all grains, neglecting local variations. Zero-field-cooled (ZFC) thermo-magnetization curves, recorded at an applied field of µ_0_*H* = 0.01 T, were used to determine points of magnetic transition of the recording medium (red line in Figure 2b). A good working point would be around 850–900 K where the coercivity basically vanishes and the medium is particularly susceptible to an applied magnetic field, although the grains retain their magnetization if no field is applied, which is not the case for temperatures ≥ *T*_C_.

## Results

**Figure 3:**
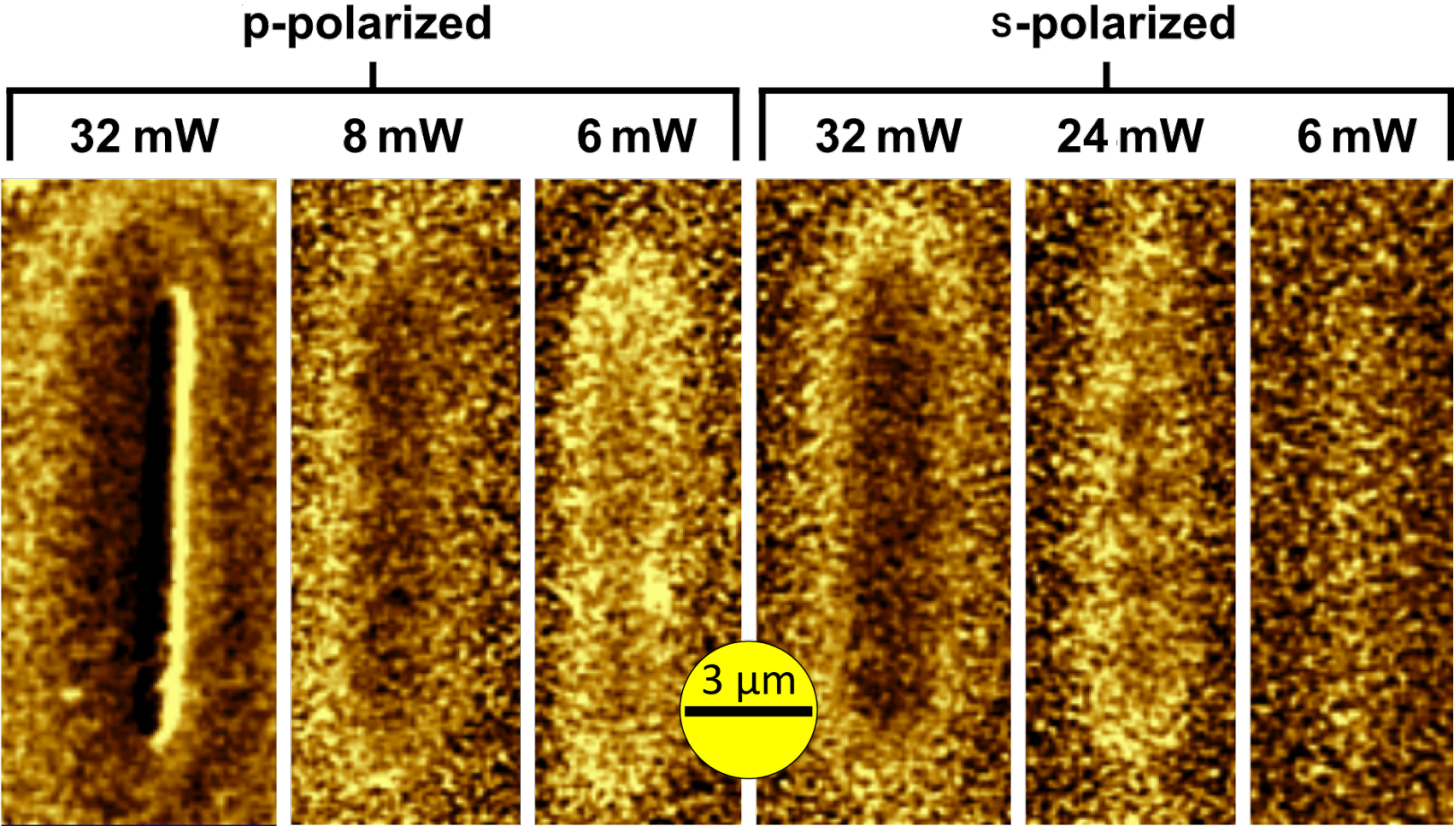
Polarization dependence of absorptivity (λ = 785 nm): p-polarized light is 3.5–4 times better absorbed than s-polarized light. Large powers damaged the sample irreversibly by laser ablation, leaving a topographical trench (not shown) and strong artefacts in the magnetic scans (see left line) where the MFM phase signal dropped three times more than for all other presented scans. The scale bar is chosen to be 3 μm, the size of the laser beam, to show the correlation between features and illumination.

A pivotal parameter in grazing-incidence HAMR is polarization. The extreme cases of p- and s-polarized incident laser light were investigated as presented in Figure 3. The AFM was set to scan a 1 × 10 µm^2^ region, with the laser shining in continuous wave (CW) mode for the duration of the scan, which took around 20 seconds. p-Polarized light got absorbed around 3.5 to 4 times better than s-polarized light (compare, e.g., the 8 mW p-pol. feature to the 32 mW s-pol. feature in Figure 3). The damage threshold for the magnetic coating lay at ~30 mW (CW illumination), which was used for creating topographical markers for AFM and optical microscopy (see left line in Figure 3). Note, both exciting wavelengths (532 nm and 785 nm) yielded basically identical results in terms of power dependence.

**Figure 4:**
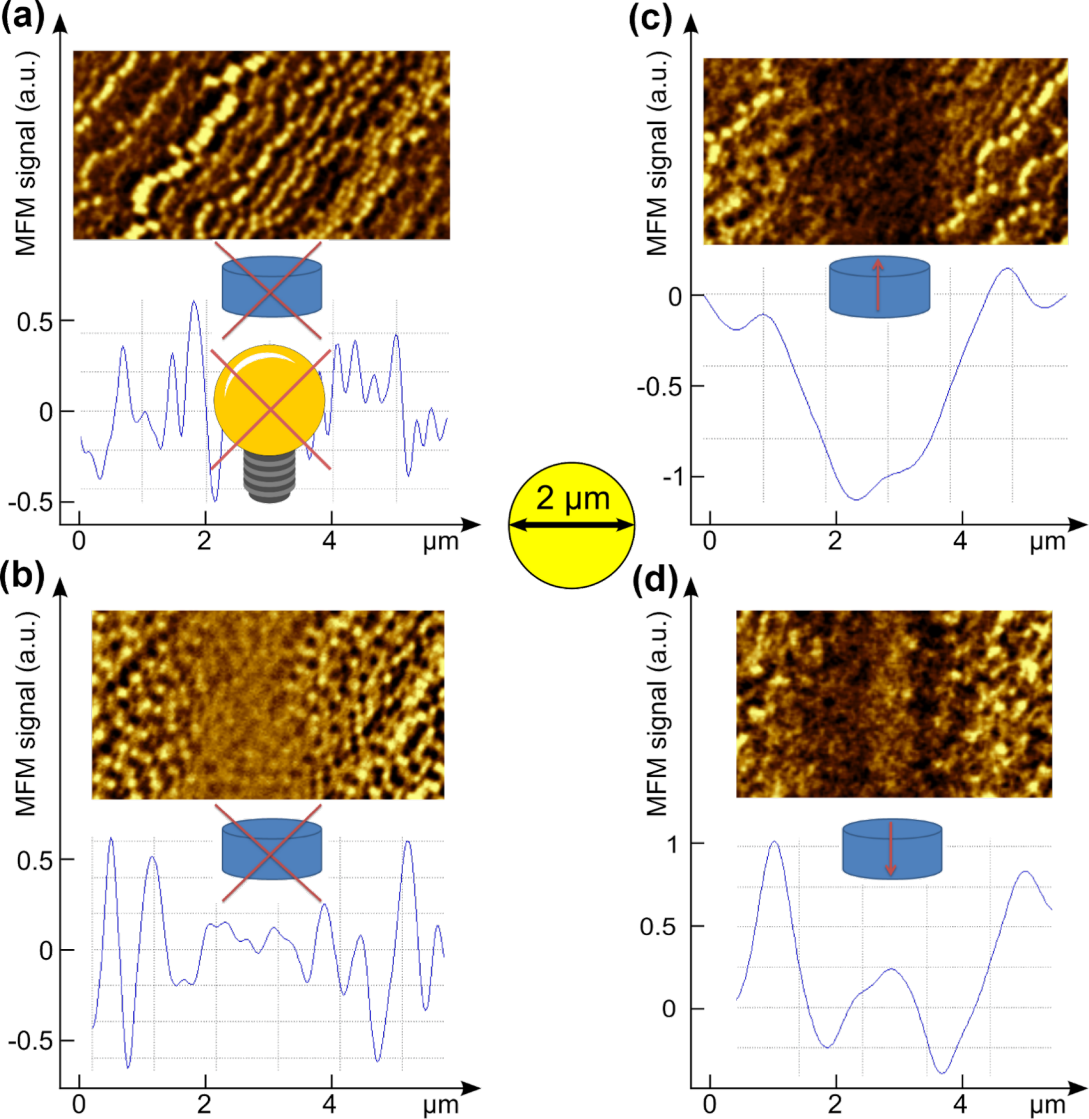
Influence of the DC bias field during laser illumination (λ = 532 nm, CW, *P* = 8 mW) on an as-is HDD piece. (a) Reference of the sample without bias field or laser irradiation. (b) Without a bias field the laser simply deletes the data and the intensity is averaged. (c) In the presence of a bias field the magnetization of the grains aligns parallel, i.e., the bias magnet’s field pointing upward yields an intensity trench. (d) If the field vector points downward there is a distinct inner hill. The scale bar is chosen to be 2 μm to show the correlation to the laser beam, similar to Figure 3.

To understand the influence of the bias field, a sample piece was left as is (with data tracks still written on it, see Figure 4a) and illuminated with p-polarized laser light at 532 nm in CW mode. In the absence of a bias field the laser simply “deleted the data”, i.e., ordered pitches turned into random noise with average intensity in the MFM phase scan image as shown in Figure 4b. In the presence of a bias field the sample magnetization aligned parallel and thus, depending on the bias field orientation, the laser-heated areas had their magnetization pointing up or down, which translates into a signal intensity drop (forming a distinct trench as in Figure 4c) and an intensity increase (forming a ridge as in Figure 4d), respectively. Note, the ridge’s signal intensity is lower than the surrounding peak intensities, since these are written under a more than 5 times stronger, highly localized (i.e., high-gradient) magnetic field. The same applies to the trench’s signal intensity in Figure 4c, which is higher than the lowest intensities in the recorded tracks. Note, details of the remnants of individual bits, which are still visible in the MFM scans, are mostly not visible in the line-profiles below because of running average performed on the graphical data.

**Figure 5:**
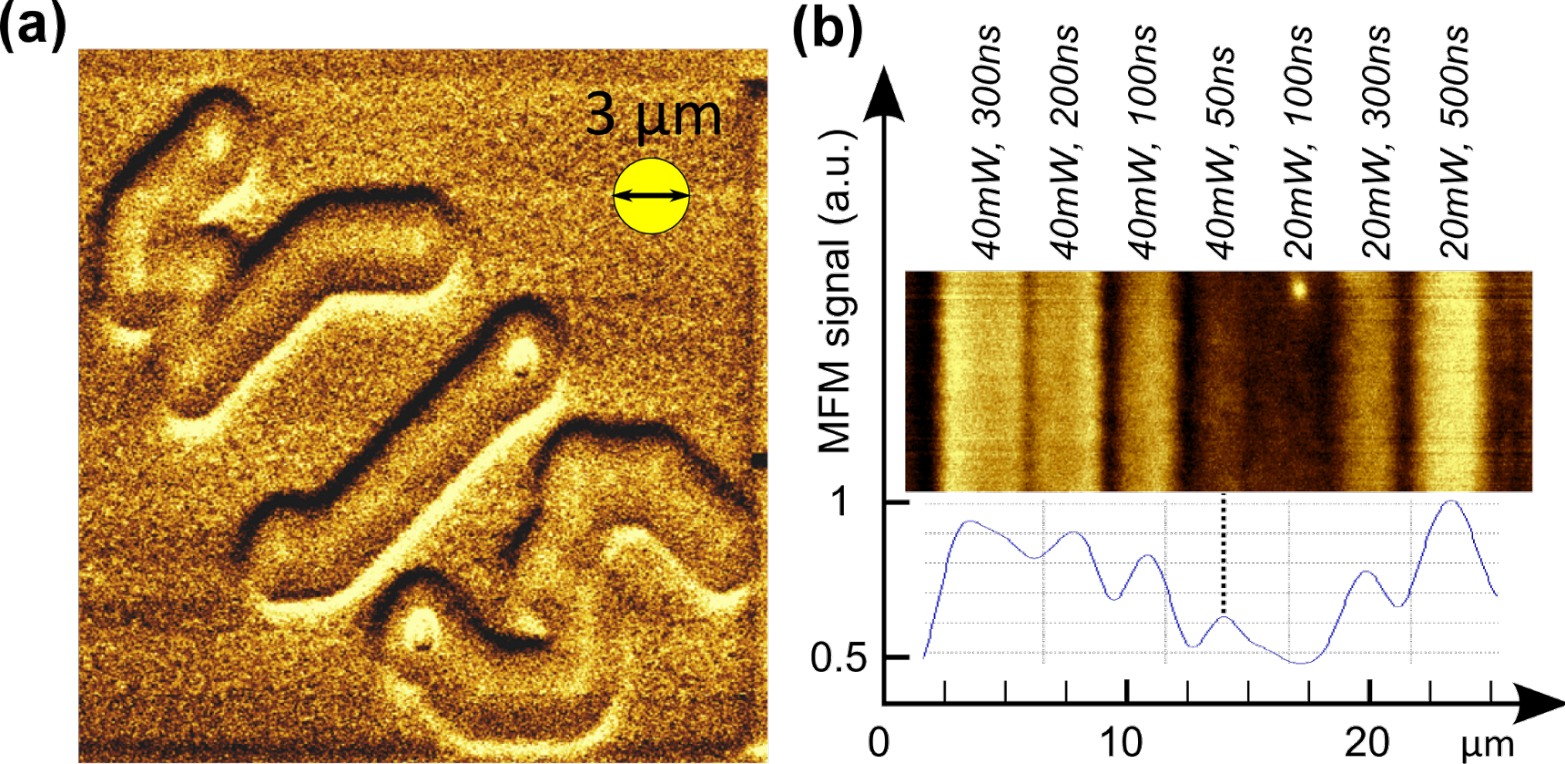
(a) Free-hand writing of magnetic features at CW illumination (p-polarized, 785 nm, 12 mW), yielding lines of around 3 μm in width – comparable to the laser spot size. (b) Line scans (p-polarized, 785 nm, powers and pulse duration as indicated) for identification of the switching power threshold (dotted line). The scale bar in (a) is chosen to be 3 μm to show the correlation to the laser beam, as in Figure 3.

Operating at CW laser illumination allowed for “free-hand” writing of magnetic features as demonstrated at laser powers of 12 mW in Figure 5a. However, the line-width resolution is limited to the μm-regime and in the example it is slightly larger than the short axis laser beam diameter. And even for lower powers (as in Figure 3) the line width is still at least 2–3 μm due to lateral heat diffusion. To find the switching power threshold, individual magnetic features in form of lines were written at variable pulse durations as depicted in Figure 5b. To create these lines, the laser (λ = 785 nm) was focused next to the AFM tip and was triggered to fire one pulse for each tip descent and a sample area of 10 × 1 μm^2^ was scanned at a resolution of 10 pixels (descents) per 1 μm. The switching threshold was found to be at 40 mW laser power and 50 ns pulse width. Due to the timescale of the heating process, thermal diffusion plays a significant role and results in lowered peak temperatures for longer pulses at smaller powers. Therefore, no magnetic feature could be detected for 20 mW/100 ns pulses, which theoretically deliver the same energy as 40 mW/50 ns pulses. Instead, the switching threshold pulse duration for 20 mW was around 150–200 ns (not shown) with a line-width broadened by thermal diffusion. To obtain the smallest possible features, large powers and short pulses are the route to success, whereas the power of the lasers used in this study was limited to 40 mW.

**Figure 6:**
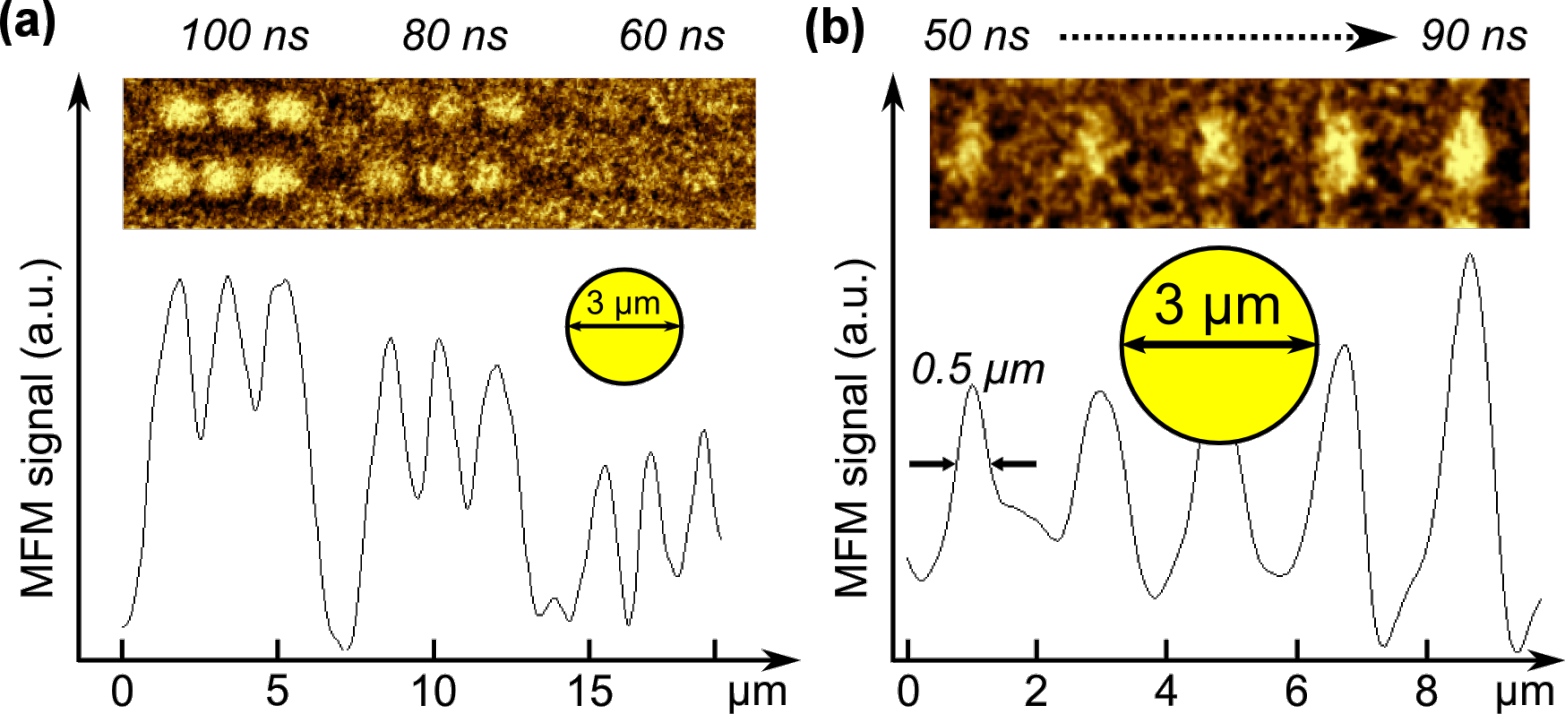
MFM phase scans and intensity profiles of “dots” laser-written at 785 nm. The laser power was kept constant at 40 mW for each feature and the energy delivered per pulse was adjusted by altering the pulse duration. (a) A series of pairs of three dots, written at pulse durations of 60–100 ns. (b) Smallest features were observed for 50 ns pulses, with lateral expansion of around 500 nm. Note, (a) and (b) were imaged in two-pass mode with different MFM tips, whose individual sensitivity dictated the image quality, thus features in (b) appear stronger than in (a). The laser focus, slightly different for each image, might also contribute to differences in the feature intensity, as well as standard AFM image processing. The scale bars in (a) and (b) are chosen to be 3 μm to show the correlation to the laser beam, as in Figure 3.

To determine the resolution limit, discrete magnetic features in form of dots were written at individual pulses of variable durations *τ* = 100, 80 and 60 ns, as in Figure 6a. The incident laser power was kept constant at 40 mW. For longer pulse durations the magnetic features were clearer but also increased in lateral dimension up to a FWHM of 1.5 μm for 100 ns pulses. The shortest possible pulse duration was found to be 50 ns, shown in Figure 6b, producing features of around 500 nm in diameter – six times smaller than the laser spot size of 3 μm (short axis). For shorter pulses the background noise became too large to identify individual features clearly.

Individual dots were well-separable in the MFM scans and well below the laser spot size. Even laser powers larger than the threshold (e.g., 100 ns pulses as in Figure 6a on the left) allow for writing multiple (4 in the example) clearly distinguishable dots in the area of the original laser spot – i.e., super-resolution.

All experiments were first conducted in the absence and then repeated in the presence of an AFM tip – yielding identical results: the tip’s presence at the sample surface (distances below 20 nm down to contact) in the center of the laser spot had virtually no impact on the heat-assisted written magnetic feature. Three different tip types were used: plain Si tips, Au-coated Si tips and MFM tips with low magnetic moment (CoCr coating). All featured a geometry which allowed for easy laser access. This was a rather surprising result as one would at least expect some sort of “hot tip” effect [15–16], increasing heating locally.

## Discussion

To develop an understanding of the underlying mechanisms which govern the heating process of the recording medium, electrical field and thermal models were created. The former to investigate the laser-material interaction, particularly the question how much laser power is absorbed; and the latter to investigate how the hot spot’s temperature and shape develop over time.

Electric field modelling was performed with Lumerical^TM^ FDTD Solutions to determine the absorption efficiency within the magnetic stack comprised of 3 nm DLC, 12 nm Co, 20 nm RuO_2_, and 30 nm Fe on a glass substrate as in Figure 2a. The stack was illuminated with a beam of a wavelength of 400–900 nm at an incident angle of γ = 70°, to allow for evaluation of practical maximum absorption efficiencies. The absorption was measured within a 50 nm square region through the full depth of each material layer for both p- and s-polarized illumination.

**Figure 7:**
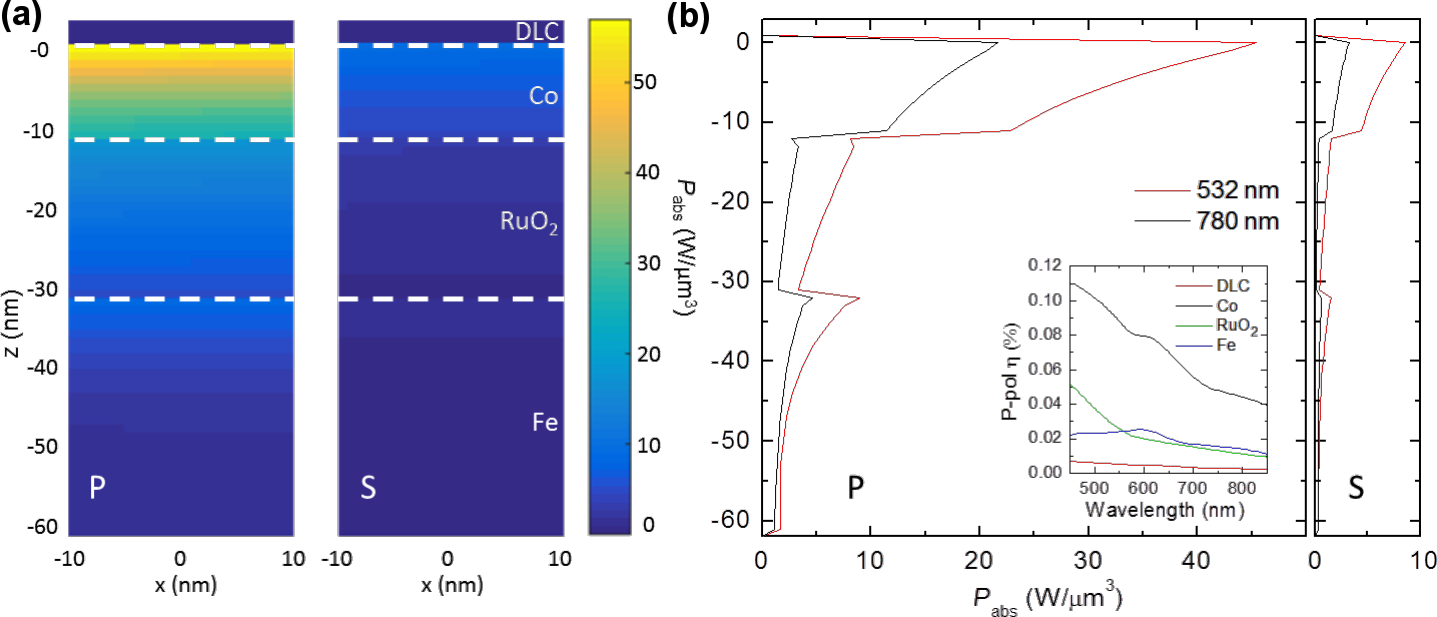
Power absorption per volume within the magnetic stack. (a) Cross-sectional absorption per layer of p- (left) and s-polarized light (right) at 532 nm. (b) Graph of absorption values in the illumination spot center axis for each polarization. Inset: Spectral absorption efficiency within a 50 nm square window integrated through the full z-extent of each medium.

Figure 7 shows the field penetration into the metal at two laser emission wavelengths – 532 and 780 nm. There is a pronounced enhancement in p- relative to s-polarized absorption for both wavelengths (by a factor of 5.3 at 532 nm). The general absorption trend still follows a simple Beer–Lambert absorption pattern for each individual layer, as depicted in Figure 7b, while maintaining a comparable power delivery to normal incidence (~70%).

The Co layer is the single greatest absorber in the stack by a factor of 2 (Figure 7, inset), which is ideal for energy transfer to the desired magnetic layer only. The inset further shows the ideal wavelength within the visible regime to be as short as possible for maximal coupling efficiency; however the greatest selective power delivery to the Co layer occurs at 625 nm while still maintaining relatively high magnetic layer absorption [17].

Using this integration region with Co, 59 fJ are required to reach a temperature of 850 K. A 100 ns duration diffraction limited beam at 532 nm will thus require sub-mW (0.56 mW) power to switch. To account for thermal losses within the system (such as diffusion into adjacent elements) we increase the effective switching threshold by up to an order of magnitude as a conservative guess, giving a resultant threshold of 5.6 mW, which is in line with the minimum power for writing seen in Figure 3. It is important to remember that the model is a simplified version of the recording material stack, thus omitting higher-order resonances, and that the Co film actually represents a layer of CoCrPt grains in a SiO_2_ matrix with significantly less efficient absorption.

Thermal modelling was done in Comsol Multiphysics^TM^ Version 5.2, using the heat module and the “Deposited Beam Power” feature. A loss of 5% at the DLC surface due to reflection was assumed, describing the case of p-polarized incident light, for which DLC has a particularly low reflectance at 70° incidence [18]. The model corresponded to the stack setup in Figure 2a.

**Figure 8:**
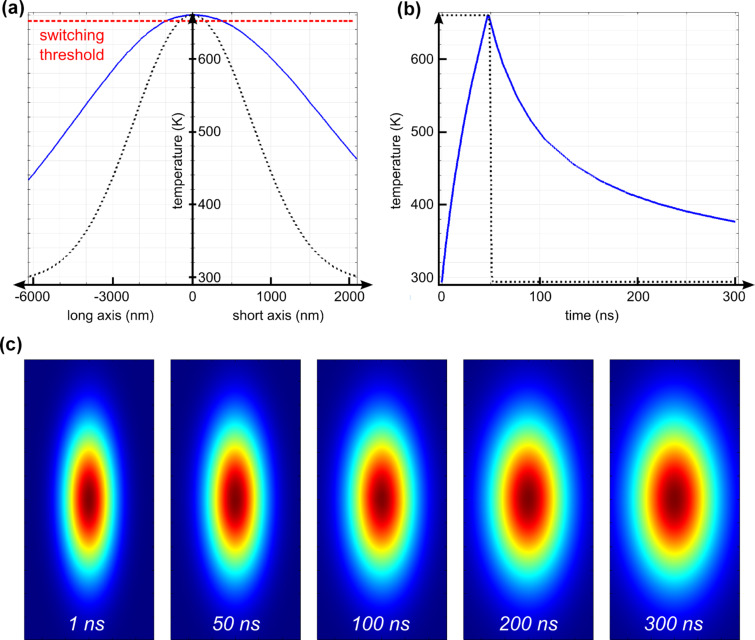
Comsol thermal modelling of a Gaussian laser pulse (*P* = 40 mW; λ = 785 nm; *τ* = 50 ns, γ = 70°) being absorbed by a thin film stack as in Figure 2a (absorption efficiency: 95%): (a) Temperature of the magnetic layer (Co) as a function of the radius (starting from the center) at the end of the 50 ns pulse. The left side of the graph shows the long axis of the elliptical hot spot and the right side shows the short axis (note, there are different scales for the horizontal axis). The intensity distribution of the laser pulse (dotted black line) defines the lateral extension of the temperature profile, which is broadened by heat diffusion. (b) Temperature at the center (*r* = 0) of the magnetic layer over time. A maximum temperature of *T_max_* ≈ 660 K is reached at the end of the pulse (dotted black line). (c) Shape of the hot spot: with increasing time the aspect ratio decreases due to lateral heat diffusion.

Figure 8a shows the lateral temperature profile in the magnetic layer (Co) relative to the Gaussian beam. A laser power of 40 mW and a pulse duration of 50 ns was chosen to correspond to the experiment. The time *t* = 0 denotes the beginning of the laser excitation, which was represented by a two-dimensional elliptical Gaussian in space and a step function in time (with a rise- and fall-time of 3 ns). The blue line denotes the temperature profile at the end of the laser pulse, i.e., *t* = 50 ns – the point of maximum temperature, and the beam intensity profile is plotted in black (dotted). It is apparent that the thermal profile follows that of the beam intensity, however, is significantly broadened by lateral heat diffusion. The switching threshold (dashed red line) is crossed at a distance from the beam center of ~1000 nm and ~300 nm for the long axis and the short axis of the elliptical hot spot, respectively, in reasonably agreement with our experimental findings. Figure 8b shows how the temperature of the magnetic layer (Co) develops over time. Under laser illumination (dotted black line) the temperature ramps up quickly to a maximum value of around *T_max_* = 660 K. After the laser pulse ended, the system cools down within a couple of hundred nanoseconds. Comparable heat modelling for alternative HAMR systems suggest a cooling time of less than 300 ns (back to room temperature), however, the system presented here does omit the cooling effect due to a large heat convection of a spinning disk and conventional PMR media lacks the benefit of an optimized heat sink structure below the magnetic layer as for FePt recording media [19–20]. Figure 8c gives the development of the shape of the hotspot: with time the hot spot becomes more circular, similar to experimental findings as in Figure 6. However, the timescale of this process in the model appears to be larger than in the experiment – the corresponding shape should be the one at *t* = 50 ns in which the hot spot still maintains a high lateral aspect ratio. The two most plausible reasons for this discrepancy are i) a stronger lateral heat diffusion in the experimental case (the model assumes isotropic heat diffusion with material constants applicable to macroscopic bodies) and ii) the dynamics of the magnetic switching process. The latter point, however, is less likely to be the cause: switching dynamics of nanoscopic ferromagnetic grains in the absence of laser heating, as in PMR media, are typically ~10 ns and heat-assistance is known to speed up the magnetic reversal process significantly [21].

It is a noteworthy observation that, although light is incident under a grazing angle, the absorption efficiency is near 100% simply by choosing the right polarization – making oblique illumination an even more efficient heating method than those geometries featuring normal illumination [13].

## Conclusion

The manipulation of the local magnetization of PMR media via a SPR-free grazing-incidence HAMR approach has been demonstrated at a resolution several times lower than the laser spot size. In a moderate magnetic bias field of μ_0_*H*_bias_ = 0.3 T magnetic features sized 500 nm – i.e., six times smaller than the laser spot – were created by single 40 mW/50 ns laser pulses on consumer-grade HDDs featuring CoCrPt-SiO_2_ PMR media. The presence of an AFM tip in the laser focus, a paradigm for other nanoscopic objects such as NFTs, did not impact on the written magnetic features at all.

Although grazing-incidence SPR-free HAMR cannot compete with alternative HAMR concepts in terms of thermal gradient [12], it opens two alternative routes for long-lifetime HAMR. First, it could be used to aid conventional HAMR (featuring a NFT) by pre-heating the recording medium and thus easing the SPR power delivery requirements, concomitantly improving the write head durability. Second, it can be used on its own to lower the coercivity to a point where a write pole with a large magnetic gradient can write features much smaller than the hot spot at moderate field strengths [22] – thus limiting the risk of cross-track erasure. This might not be satisfactory for high-end applications which employ many random writes, but certainly becomes a viable alternative for cold data storage, as in latest-generation shingled magnetic recording (SMR) devices, which allow for a storage capacity increase of up to 50% compared to conventional PMR. In SMR data is not written in isolated parallel tracks, but in bundles of overlapping tracks, where changes in one track require a renewal of the whole bundle in any case [23]. Current single-pole write heads could be upgraded to host several write poles working in parallel for improved speed and better exploitation of the comparatively large hot spot. A pivotal prerequisite for this approach would be a large magnetic field gradient of the write pole as in PMR.

With appropriate optics and the resulting improved laser focus, not only could the hot spot size be shrunk significantly, but also the power density at the recording disk could easily be increased by a factor of 10, which enables the use of low-power laser sources and pulses shorter than 20 ns, comparable to industry HAMR benchmarks [21]. Power requirements are further eased by using FePt recording media [24], designed for improved laser light absorption and lower operating temperatures.

Besides considerations of durability, SPR-free HAMR also opens new possibilities for designing HDD write heads, since laser illumination from the side does not require sophisticated integrated photonics and could draw power from one common laser source, conveniently delivered to multiple write heads through optical fibers. This will help implement larger light sources, which cannot be integrated in a recording head, such as ultra-short pulsed lasers [13].
